# *EI24* regulates epithelial-to-mesenchymal transition and tumor progression by suppressing TRAF2-mediated NF-κB activity

**DOI:** 10.18632/oncotarget.1434

**Published:** 2013-11-17

**Authors:** Jung-Min Choi, Sushil Devkota, Young Hoon Sung, Han-Woong Lee

**Affiliations:** ^1^ Department of Biochemistry, College of Life Science and Biotechnology, Yonsei University, Seoul 120-749, Korea; ^2^ Laboratory Animal Research Center, Yonsei University, Seoul 120-749, Korea

**Keywords:** EI24, EMT, Tumor progression, NF-κB

## Abstract

Tumor metastasis is a multistep process that requires the concerted activity of discrete biological functions. The epithelial-to-mesenchymal transition (EMT) is the most critical mechanism implicated in tumor progression that is controlled by the inflammatory microenvironment. Understanding how an inflammatory microenvironment is maintained and contributes to tumor progression will be crucial for the development of new effective therapies. Here, we report that *etoposide induced 2.4 (EI24)* has a multifaceted role against tumor progression that is regulated by both EMT and inflammation. Decreased expression levels of EI24 in epithelial tumor cells induced EMT in association with increased cell motility and invasiveness and resistance to anoikis. Overexpression of EI24 resulted in the opposite cell biological characteristics and suppressed in vivo metastatic behavior. EI24 attenuated NF-κB activity by binding to the Complex I component TRAF2 and inducing its lysosome-dependent degradation, leading to transcriptional alterations of EMT-and inflammation-related genes. Analysis of clinical samples demonstrated that reduced EI24 expression and copy number was positively correlated with tumor malignancy and poor prognosis. Collectively, these findings establish EI24 as a critical suppressor of tumor progression and implicate EI24 expression level in malignant tumors as a useful therapeutic and diagnostic marker.

## INTRODUCTION

Tumor metastasis, the spread of cancer cells from the primary neoplasm to distant organs, is a sequential and multistep process that is the most common cause of death in cancer patients [1]. Malignant tumor cells acquire several traits including increased cell motility, loss of cell-to-cell adhesion, invasion into lymphatic and/or blood circulation, and resistance to anoikis [2]. To acquire malignancy, cancer cells must undergo a loss of epithelial phenotypes and acquire characteristics of a mesenchymal state. This process, called the epithelial-to-mesenchymal transition (EMT), changes many of the cellular properties described above [3].

NF-κB mediates molecular crosstalk between inflammation and many other physiological processes through the transcriptional regulation of pro-inflammatory genes [4]. The transcriptional activity of NF-κB is controlled by Complex I of the tumor necrosis factor receptor type 1 (TNFR1) signaling pathway, which contains TNFR1-associated DEATH domain protein (TRADD) and TNFR-associated factors 2/5 (TRAF2/5). In the canonical pathway, IκBα is phosphorylated in an IKKβ-and NEMO-dependent manner, resulting in the nuclear translocation of heterodimers containing NF-κB p65 [5]. Thus, inhibition of IKKβ/NF-κB signaling could be an effective tool for the inhibition of tumor progression through reduced expression of a number of pro-malignant genes [6, 7].

The pro-apoptotic gene *EI24* plays an important role in the negative regulation of cell growth through its tumor suppressor activities [8]. The genomic locus of *EI24* at chromosome 11q23-q24 is a hot spot region for mutations in human tumors and is often correlated with poor prognosis [9]. Additionally, loss of EI24 expression is associated with the development of invasive ductal [10] and cervical [11] carcinomas. However, the involvement of EI24 in tumor malignancy and the underlying molecular mechanisms are not well characterized.

In this study, we examined the functional significance of EI24 in the regulation of EMT and tumor progression by investigating the properties of cancer cells in which EI24 was either overexpressed or downregulated and the physiological activity of these cells in a mouse model of cancer. We found that, mechanistically, EI24 attenuated NF-κB activity by binding to the Complex I component TRAF2 and causing its lysosome-dependent degradation, thereby suppressing the transcription of pro-inflammatory genes that contribute to tumor progression. Furthermore, our data showed that decreased EI24 expression correlated with high tumorigenic potential of various cancer cells and with poor prognosis in human cancer patients.

## RESULTS

### EI24 inhibits cell motility and enhances cell-cell adhesion

**Figure 1:**
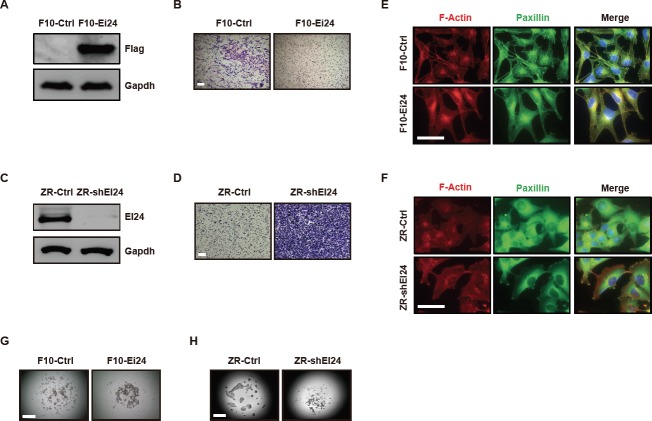
EI24 regulates the cell motile phenotype (A) and (C) Immunoblot analysis of EI24 expression in B16F10 cells expressing either control vector (F10-Ctrl) or EI24 (F10-Ei24, A), and in ZR-75-1 cells stably expressing control (ZR-Ctrl) or EI24-specific shRNA (ZR-shEI24, C). (B) and (D) Cell migration assays with F10-Ei24 (B) and ZR-shEI24 cells (D). The cells were visualized by staining with crystal violet. Data are representative of at least two independent experiments. Scale bars represent 200 μm. (E) and (F) Actin cytoskeletal structures in B16F10 (E) and ZR-75-1 (F) variant cells were visualized by immunofluorescence (IF) staining using anti-Phalloidin and anti-Paxillin antibodies. Scale bars represent 50 μm. (G) and (H) Cell-cell adhesion in F10-Ei24 (G) and ZR-shEI24 (H) cells was evaluated using the hanging drop assay. Data are representative of five independent drops.

Because increased cell motility is a key step in tumor progression [12], we first investigated the effect of EI24 on the motility phenotypes of several cancer cell lines. Overexpression of EI24 in metastatic B16F10 cells (F10-Ei24 cells) significantly reduced migration compared with that of the control cells (Figure 1A, 1B). Conversely, stable EI24 knockdown (ZR-shEI24 cells) increased cell migration (Figure 1C, 1D). Consistent with these data, F10-Ei24 cells exhibited a retarded wound-healing capacity (Supplemental Figure 1A), whereas EI24 knockdown in B16F10 cells (F10-shEi24) increased wound-healing capacity (Supplemental Figure 1B). As rearrangement of the actin cytoskeleton is an important factor in cell migration [13], we monitored F-actin arrangements and focal adhesion distribution in the context of overexpression or knockdown of EI24. The formation of stress fibers and focal adhesions was diminished in F10-Ei24 cells, whereas ZR-shEI24 cells displayed well-organized stress fibers linked with focal adhesions (Figure 1E, 1F). These data indicate that EI24 decreases cell migration by suppressing the formation of stress fibers and focal adhesions.

The adhesive strength of cells also has a critical influence on cell motility [14], therefore we measured the effect of EI24 overexpression on cell-cell adhesion. F10-Ei24 cells exhibited increased cell aggregation in hanging drop cultures (Figure 1G). A similar effect of EI24 overexpression was observed in breast cancer 4T1 cells (Supplemental Figure 1C). In contrast, EI24 knockdown significantly decreased cell-cell adhesion of ZR-75-1 and NMuMG cells (Figure 1H, Supplemental Figure 1D). These findings indicate that EI24 is necessary for maintenance of cell-cell interactions.

### Decreased EI24 expression induces EMT

**Figure 2:**
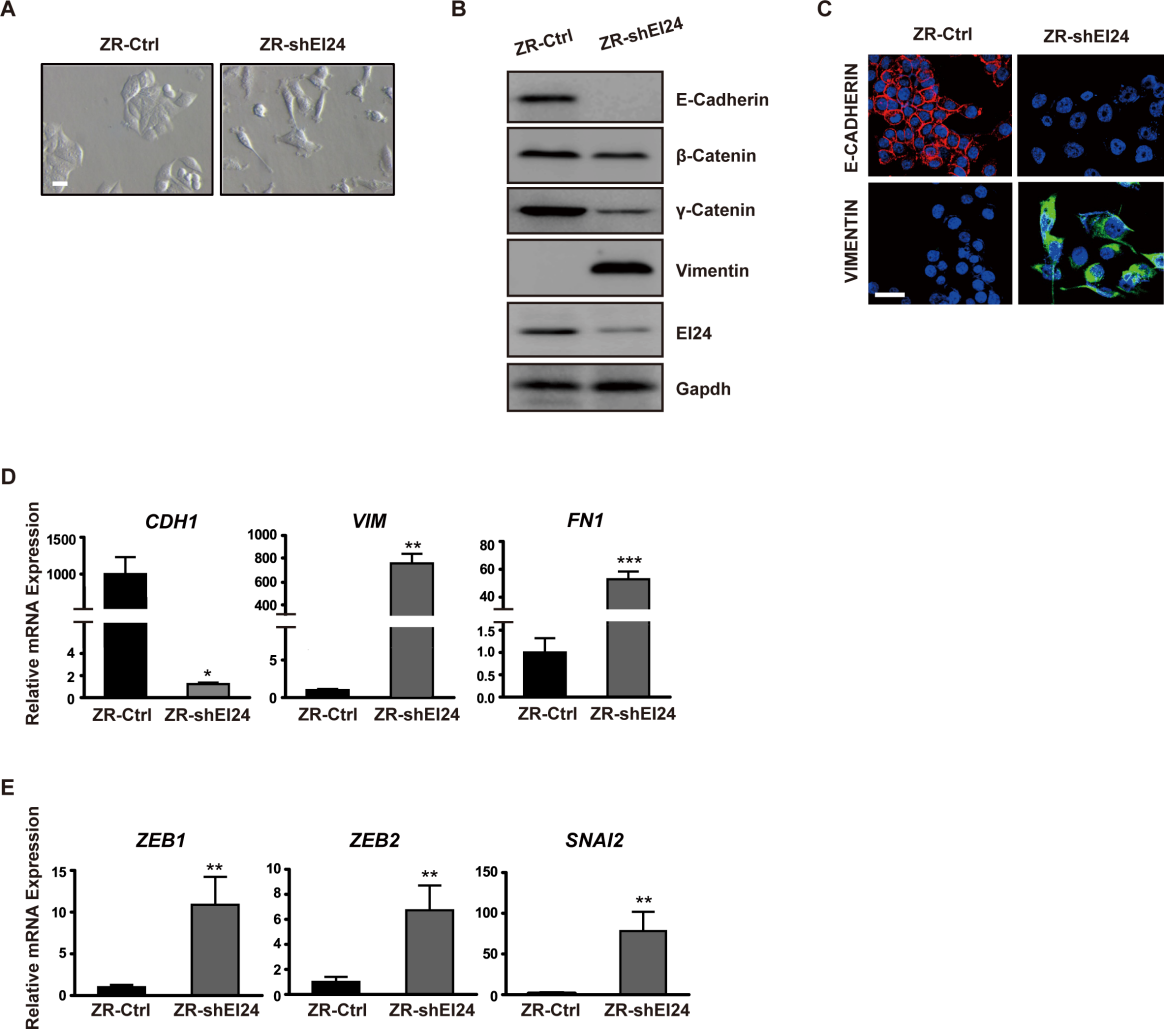
Knockdown of EI24 induces EMT (A) Phase contrast microscopy of ZR-75-1 cells stably expressing control or EI24-specific shRNA. Scale bar represents 100 μm. (B) Immunoblot analysis of changes in the expression of epithelial and mesenchymal markers in control and ZR-shEI24 cells. (C) Confocal microscopic analysis of E-cadherin and Vimentin expression in ZR-Ctrl and ZR-shEI24 cells. Scale bar represents 50 μm. (D) and (E) Real-time quantitative PCR (qPCR) analysis of transcriptional regulation of the expression of epithelial and mesenchymal markers (D) and EMT-related transcription factors (E) in control and ZR-shEI24 cells. Data are means of measurements from two independent experiments performed in triplicate; P values were calculated by unpaired t-test using GraphPad Prism software. *p <0.05, **p <0.01, ***p <0.0001.

Because the increased cell motility, cytoskeleton rearrangements, and decreased cell-cell adhesion induced by reduced levels of EI24 are reminiscent of EMT, we examined whether EI24 ablation affected the epithelial characteristics of cancer cells. Gene set enrichment analysis (GSEA) showed a strong correlation between EI24 knockdown in ZR-75-1 cells and gene signatures that are invoked during the EMT process [15-17] (Supplemental Figure 2A-C). Additionally, gene sets characteristic of phenotypic changes and molecular signaling alterations that are prerequisites for EMT were enriched in ZR-shEI24 cells (Supplemental Table 1, 2). In this context, we investigated whether reduced expression of EI24 induces EMT. Consistent with the molecular transition, EI24 knockdown induced a morphological change of ZR-75-1 cells to the fibroblast-like scattered morphology of mesenchymal cells (Figure 2A). A significant reduction in the expression of epithelial markers such as E-cadherin, β-catenin, and γ-catenin and emergence of the mesenchymal marker vimentin further supported the induction of EMT by EI24 knockdown (Figure 2B). Moreover, expression of the epithelial marker E-Cadherin in the cell-to-cell contacts was significantly decreased, coincident with increased expression of the mesenchymal marker vimentin (Figure 2C). We consistently found that the level of *CDH1* mRNA was significantly lowered whereas the mRNA levels of *VIM* and *FN1* were considerably increased in EI24 knockdown cells (Figure 2D). Notably, mRNA levels of EMT-related transcription factors including *ZEB1*, *SIP1*, and *SLUG* were also significantly increased in ZR-shEI24 cells (Figure 2E). Collectively, these data indicate that EI24 plays an important role in maintaining epithelial characteristics; thus, reduced expression of EI24 promotes the initiation of EMT.

### Reduced EI24 expression potentiates malignant properties of tumor cells

**Figure 3:**
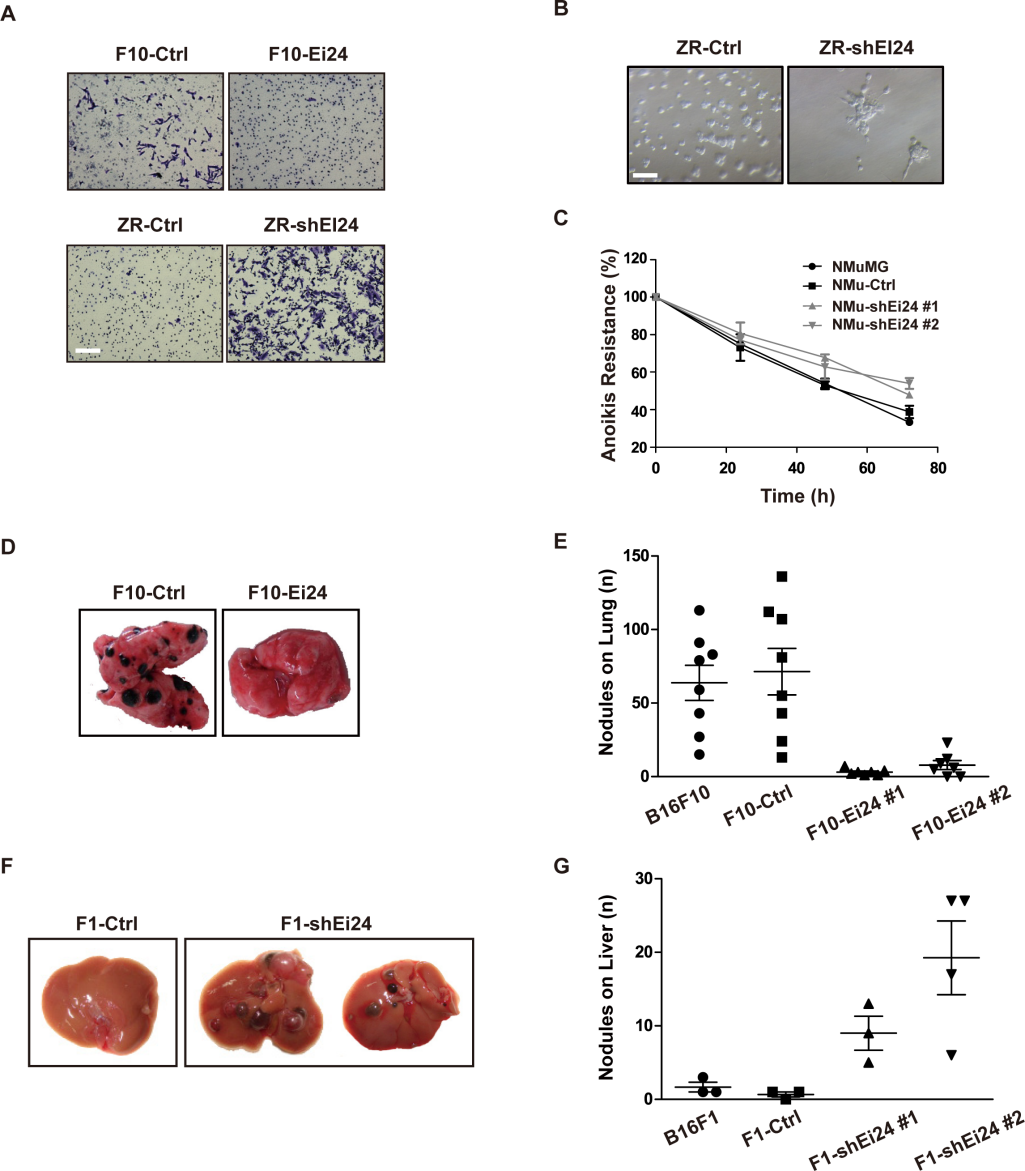
Knockdown of EI24 promotes malignant characteristics that accompany EMT (A) The indicated cell lines were added to Matrigel-coated transwell inserts, and invading cells were visualized after 16 hours by crystal violet staining. Scale bar represents 200 μm. (B) Formation of invasive acini was evaluated using the 3D matrigel assay. The data are representative of at least two independent experiments. Scale bar represents 100 μm. (C) NMuMG Parental, NMu-Ctrl and NMu-shEi24 cells were maintained in suspension using ultra-low-attachment plates and cell viability was analyzed at the indicated time points. (D) and (E) F10-Ctrl or F10-EI24 cells were intravenously injected into C57BL/6NTac mice and specific metastatic ability was analyzed by counting nodules in the lungs; n ≥7; one-way analysis of variance, p <0.0001. (F) and (G) F1-Ctrl or F1-EI24 cells were intravenously injected into C57BL/6NTac mice and their specific metastatic ability was analyzed by counting nodules in the liver; n ≥3; one-way analysis of variance, p <0.01.

Since EMT is a characteristic feature of malignant tumors, we investigated cellular properties that are accompanied by EMT in the context of EI24 dysregulation. Matrigel invasion assays revealed that EI24 overexpression inhibited the invasive capacity of malignant B16F10 cells (Figure 3A, top panel). In contrast, ZR-shEI24 cells showed increased invasiveness (Figure 3A, bottom panel). ZR-shEI24 cells in 3D-culture consistently displayed invasive characteristics compared with the rounded morphology of the control cells (Figure 3B). We next examined the effect of EI24 on anchorage independent survival by measuring the cell viability after inducing the anoikis. After 72 hours, the EI24 knockdown cells survived approximately 15% more than the control cells (Figure 3C). This result indicates that loss of EI24 protects cells from anoikis. Taken together, acquisition of invasive characteristics and resistance to anoikis upon reduced levels of EI24 facilitates the enhanced malignancy of tumor cells.

To validate our in vitro findings using in vivo analyses, we adopted a well-established mouse model of experimental metastasis by injecting B16 variant cells intravenously into congenic C57BL/6NTac mice [18]. Control B16F10 cells formed large numbers of visible metastasized nodules, whereas the formation of lung nodules was significantly attenuated in mice injected with F10-Ei24 cells (Figure 3D, 3E). When liver-colonizing B16F1 variant cells were injected into C57BL/6NTac mice [19], Ei24 knockdown increased the formation of metastatic nodules in the mouse liver, compared to the number of nodules formed by control cells (Figure 3F, 3G). Collectively, these data support the notion that reduced expression of EI24 promotes tumor malignancy in vitro and in vivo.

### EI24 inhibits NF-κB transcriptional activity and the expression of inflammation-associated target genes

**Figure 4:**
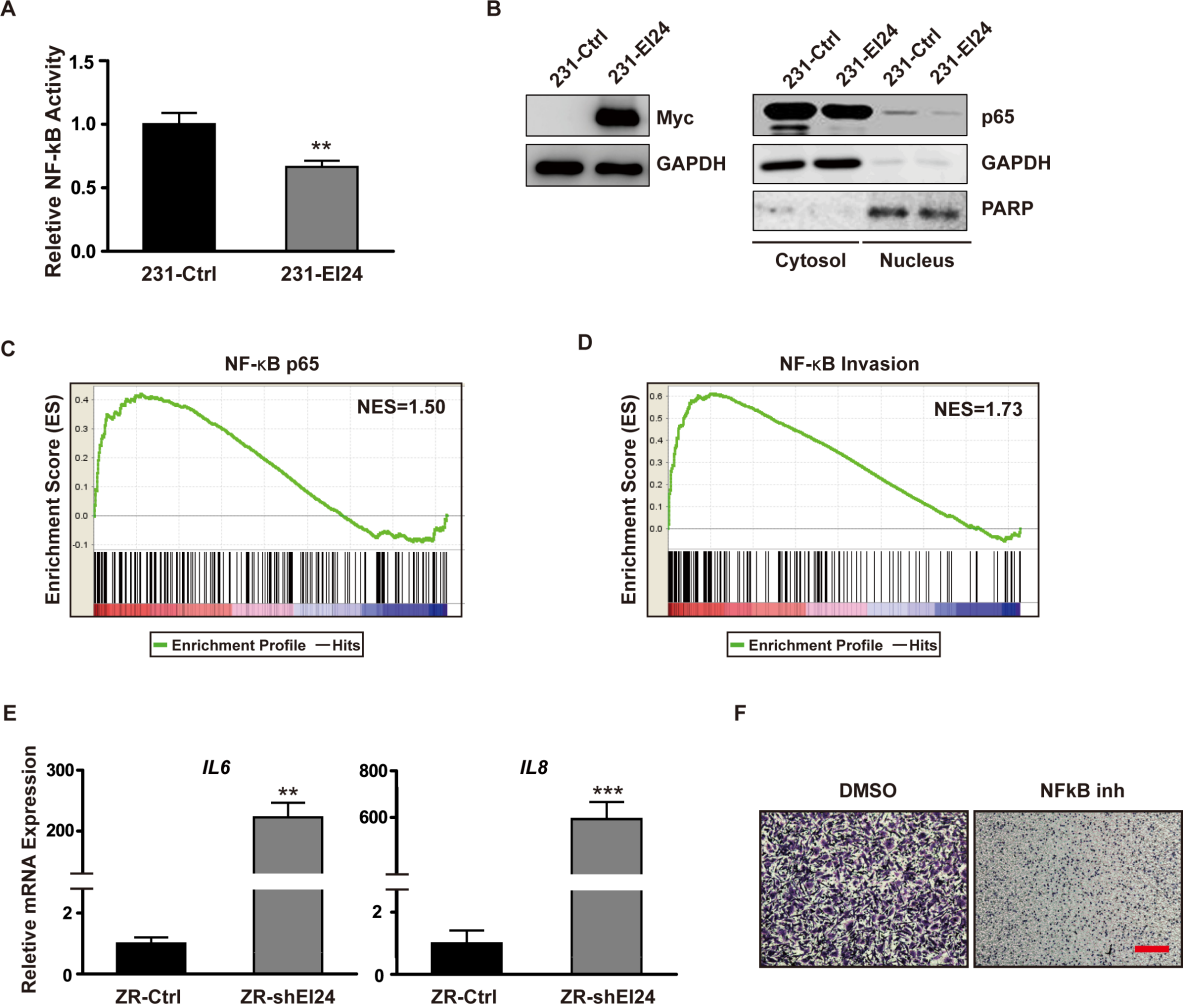
NF-κB activation and upregulation of genes involved in inflammation-induced tumor progression by EI24 knockdown (A) The transcriptional activity of NF-κB in control and EI24-overexpressing MDA-MB-231 cells was evaluated using a NF-κB luciferase reporter assay. Data shown are mean ± S.D. n = 6, **p <0.0001. (B) Immunoblot analysis of p65 expression levels in nuclear extracts from control and EI24-overexpressing MDA-MB-231 cells. (C) and (D) GSEA for gene signatures in EI24 knockdown cells indicating genes that contain p65-binding sites in their promoter regions (p <0.001; C) and genes that are targets of NF-κB and are associated with tumor invasiveness (p <0.001; D). NES, normalized enrichment score. (E) Relative mRNA expression levels of the indicated genes in control and ZR-shEI24 cells analyzed by real-time qPCR. Data shown are the mean ± S.D. of two independent experiments with each sample assayed in triplicate; P values were calculated by unpaired t-test using GraphPad Prism software. *p <0.005, **p <0.0001, ***p <0.0005. (F) Cell migratory potential was measured after treatment of ZR-shEI24 cells with an NF-κB inhibitor. The red scale bar represents 200 μm.

To elucidate the molecular mechanism governing EI24-mediated regulation of EMT, we treated ZR-shEI24 cells with specific inhibitors of several pathways. Although most of the inhibitors had no effect, the NF-κB inhibitor BAY 11-7082 restored the epithelial morphology (Supplemental Figure 3A). From these data, we surmised that EI24 suppresses EMT by negatively regulating NF-κB signaling. Consistent with this, overexpression of EI24 suppressed NF-κB reporter activity in MDA-MB-231 cells (Figure 4A). These observations were also confirmed using EI24 overexpression and knockdown systems in HeLa cells (Supplemental Figure 3B, 3C). EI24 overexpression in MDA-MD-231 cells significantly decreased nuclear localization of the p65 subunit of NF-κB (Figure 4B), whereas F10-shEI24 cells exhibited an increase in p65 in the nucleus (Supplemental Figure 3D). Furthermore, when GSEA was performed using a gene set from the Molecular Signatures Database (MSigDB) that contained the GGGRATTTCC motif, the consensus binding sequence for p65 transcription factors, in their promoter regions [20] this gene signature was enriched in ZR-shEI24 cells (Figure 4C). Notably, the expression levels of NF-κB target genes that are associated with tumor invasiveness [21] were also significantly enhanced in ZR-shEI24 cells (Figure 4D). For example, ZR-shEI24 cells exhibited upregulation of *IL6* and *IL8,* NF-κB target genes that are involved in inflammation-induced tumor metastasis (Figure 4E). Consistent with these data, *IL6* and *IL8* were downregulated in MDA-MB-231 cells upon overexpression of EI24 (Supplemental Figure 3E). Moreover, treatment of ZR-shEI24 cells with the NF-κB inhibitor BAY 11-7082 significantly decreased cell migration compared with the control treatment (Figure 4F). Taken together, these data demonstrate that EI24 attenuates NF-κB-mediated tumor malignancy.

### EI24 knockdown promotes sustained TNFR1 Complex I signaling

**Figure 5:**
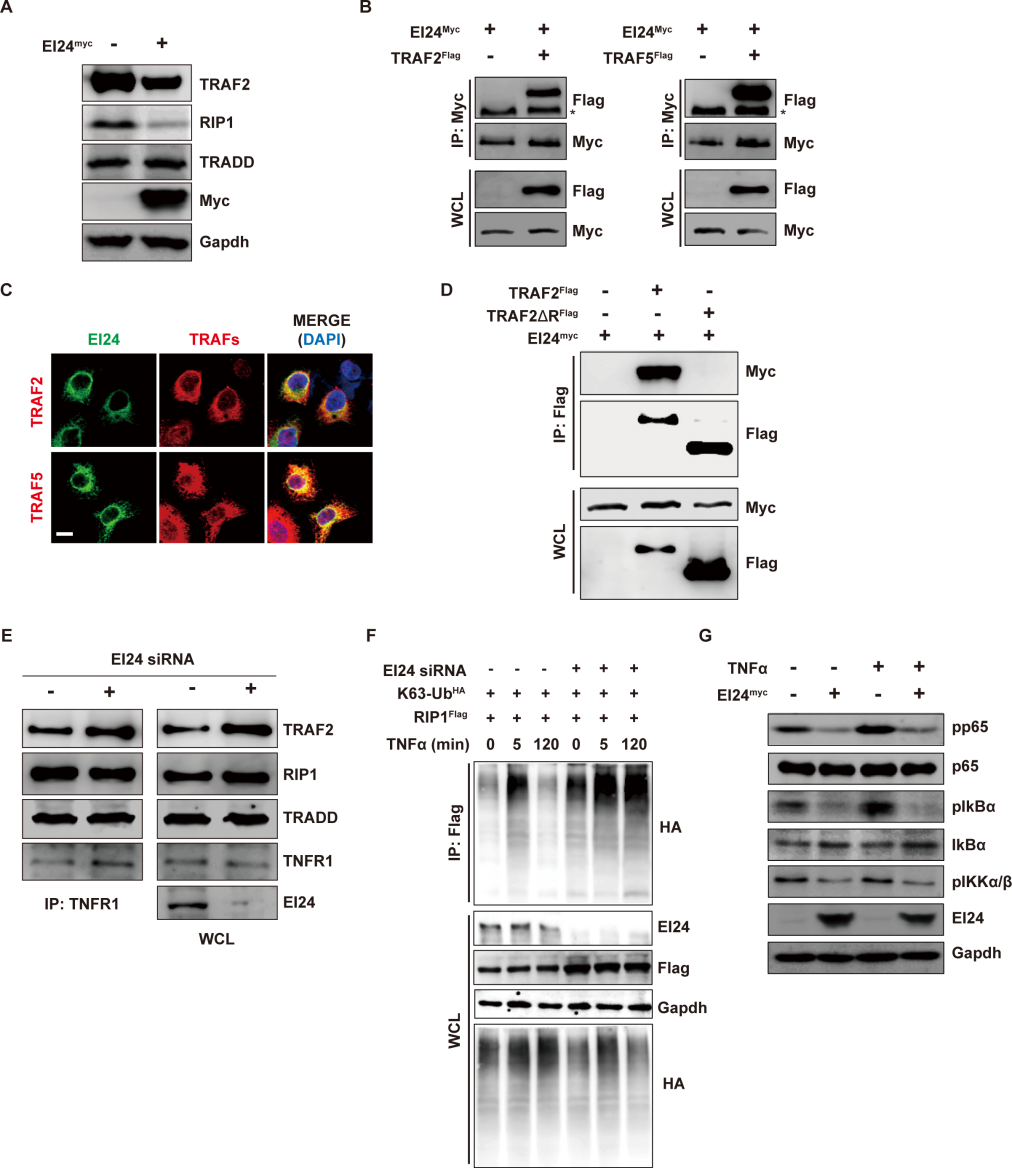
EI24 binds to and promotes degradation of Complex I components, thus inhibiting downstream signaling (A) Immunoblot analysis of degradation of TRAF2, RIP1, and TRADD induced by EI24. (B) Interaction between EI24 and TRAF2/5 in 293T cells assessed using immunoprecipitation. (C) Colocalization of EI24 with TRAF2/5 in HeLa cells. Scale bar represents 10 μm. (D) Immunoprecipitation experiments showing that the RING domain of TRAF2 is required for its interaction with EI24. (E) siRNA-mediated knockdown of EI24 increases the binding of TRAF2 to TNFR1. Binding of TNFR1 with TRAF2, TRADD, and RIP1 was examined in control and EI24 knockdown 293T cells. WCL, whole cell lysate. (F) EI24 knockdown results in accumulation of K63-linked RIP1. RIP1-K63 ubiquitination was examined in the presence of TNFα at the indicated time points. (G) Downstream molecules of Complex I-mediated signaling were evaluated upon EI24 overexpression. Levels of phospho (p)-IKKα/β, p-IκBα, IκBα, p-p65, and p65 in MDA-MB-231 cells were determined by immunoblotting.

Next, we focused on identifying upstream components of the molecular mechanism by which loss of EI24 increases NF-κB transcriptional activity. Among several pathways that converge on NF-κB, the most prominent is the Complex I (TRADD, RIP1, and TRAF2) signaling cascade induced by the binding of TNFα to its receptor [5]. We confirmed involvement of EI24 in this process by GSEA using gene signatures of the TNFα pathway acting through NF-κB [22] in EI24 knockdown cells (Supplemental Figure 4A). Because TRAF2 is known to be a mediator of inflammatory cytokine-induced NF-κB activation [23] and reduced levels of EI24 resulted in the promotion of tumor invasiveness through NF-κB (Figure 4), we examined whether the increase in NF-κB transcriptional activity upon EI24 knockdown is mediated through activation of Complex I signaling. First, we assessed changes in protein levels of Complex I components. RIP1, TRAF2, and TRAF5 were significantly downregulated by EI24 overexpression and upregulated by EI24 knockdown (Figure 5A, Supplemental Figure 4B, 4C). Based on previous reports showing that EI24 acts as an inducer of autophagy [24, 25], we examined whether inhibition of lysosome function rescues the degradation of TRAF2 and TRAF5 that is mediated by overexpression of EI24 and found that treatment with the lysosome inhibitor bafilomycin A1 impeded EI24-mediated degradation of TRAF2 and TRAF5 (Supplemental Figure 4D). This result suggests that the regulation of NF-κB activity by EI24 is mediated through lysosomal degradation of Complex I components.

Since TNFα-induced NF-κB signaling in mice is impaired only when *Traf2* and *Traf5* are deficient [26], it is plausible that the simultaneous downregulation of TRAF2 and TRAF5 by EI24 results in activation of the NF-κB pathway. To examine whether the degradation of TRAF2 and TRAF5 by EI24 is mediated through a physical interaction between these proteins we performed immunoprecipitation and immunofluorescence assays in 293T and HeLa cells and showed that EI24 bound to and colocalized with both TRAF2 and TRAF5 (Figure 5B, 5C, Supplemental Figure 4E). Domain mapping assays revealed that the Really Interesting New Gene (RING)-domain of TRAF2 facilitates the binding of TRAF2 with EI24 and the degradation of TRAF2 (Figure 5D, Supplemental Figure 4F). Furthermore, knockdown of EI24 resulted in increased binding of TRAF2 to TNFR1 without affecting the interaction between RIP1 and TRADD in Complex I (Figure 5E). These data indicate a role of EI24 recruitment and degradation of the components of Complex I in the alleviation of TNFR1 signaling.

TRAF2 is a key component of NF-κB signaling through its role as an RING-domain-containing E3 ubiquitin ligase that mediates the K63-linked polyubiquitination of RIP1 [27]. In turn, K63-linked polyubiquitinated RIP1 functions as a scaffold for the assembly of factors that activate IKK in NF-κB activation-dependent inflammatory signaling pathways [28]. Because knockdown of EI24 resulted in the accumulation of TRAF2, we examined the K63-ubiquitination status of RIP1. After treatment with TNFα, K63-linked ubiquitination of RIP1 was sustained for 2 hours in EI24 knockdown cells but was almost completely absent in control cells at the same time point (Figure 5F). These data imply that in the course of signal transduction a reduction in the EI24 level plays an important function in sustaining activation of downstream NF-κB signaling by maintaining K63-ubiquitination of RIP1. Consequently, the downstream molecules of Complex I accumulated upon EI24 knockdown, resulting in activation of IKKβ, IκBα, and p65 (Supplemental Figure 4G). We also verified that the TNFα-mediated activation of NF-κB was significantly suppressed by ectopic EI24 expression in MDA-MB-231 cells (Figure 5G). Conversely, knockdown of EI24 augmented TNFα-mediated NF-κB activation (Supplemental Figure 4H). Taken together, these data demonstrate that EI24 acts as a negative regulator of TRAF2 signaling by degrading Complex I components, leading to decreased NF-κB transcriptional activity.

### EI24 expression is inversely correlated with tumor invasiveness and poor prognosis in human patients

**Figure 6:**
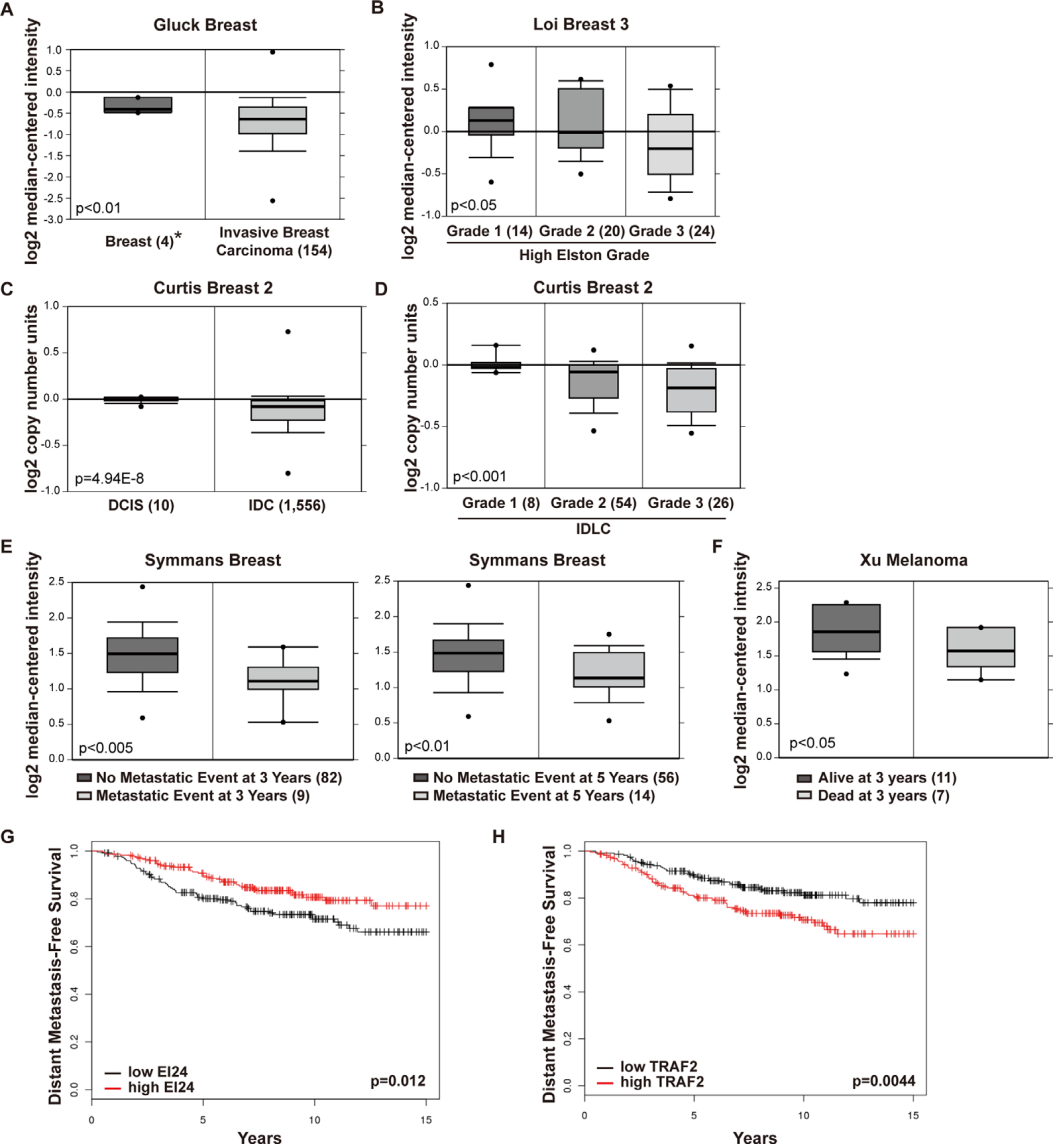
Decreased expression of EI24 correlates with increased tumor invasiveness and poor prognosis in human patients (A) Box plots showing EI24 gene expression in normal breast tissue and invasive breast carcinoma of breast cancer patients. (B) Box plots showing EI24 gene expression in high Elston-Ellis grade breast carcinomas. (C) and (D) Box plots showing EI24 gene copy numbers in DCIS and IDC (C), and in IDLC of various tumor grades (D). (E) EI24 gene expression in patients with breast cancer and its association with clinical outcomes at 3 and 5 years in patients with one or more metastatic events. (F) EI24 gene expression in melanoma patients and clinical outcomes at 3 years. (G) and (H) Kaplan-Meier plots of distant metastasis-free survival of human breast cancer patients. ER-positive patients (n = 417) were evaluated from 2011 version of database and categorized by median expression of EI24 (probe set 208289_s_t, G) or TRAF2 (probe set 204413_at, H). The p-values were calculated using the log-rank test. *Numbers in parentheses indicate number of samples included in the study.

To validate our findings from cell lines and mouse models in human patients, we searched publicly accessible microarray datasets for correlations between EI24 expression and clinically relevant parameters [29]. The Gluck breast dataset [30] showed markedly reduced *EI24* mRNA expression in invasive breast carcinomas compared with that in normal breast tissues (Figure 6A). In a dataset from The Cancer Genome Atlas (TCGA), EI24 expression was downregulated in invasive ductal breast carcinomas (IDCs) and invasive lobular breast carcinomas (ILCs) compared with normal breast samples (Supplemental Figure 5A). Furthermore, EI24 expression correlated negatively with the progression stage of breast cancer (Figure 6B) measured using the Elston-Ellis grading system [31]. Consistent with the EI24 expression pattern, the copy number of the *EI24* gene was decreased in IDC [32] compared with ductal carcinoma in situ (DCIS, Figure 6C). Furthermore, *EI24* copy number loss correlated with high tumor grade in IDC and ILC (Figure 6D). We also detected loss of *EI24* copy number in the most common types of invasive breast cancer, such as IDC, ILC, and mixed ductal/lobular carcinoma (IDLC), in the TCGA database (Supplemental Figure 5B). Similar *EI24* gene expression patterns were observed in metastatic tumors from various types of cancers (Supplemental Figure 5C). These findings demonstrate that the expression level and copy number of the *EI24* gene might be good prognostic markers in breast cancer patients.

Based on these data, we analyzed the correlation between EI24 expression level and clinical outcomes in patients with breast tumors. In the Symmans 2 and Loi breast cancer datasets [33, 34], estrogen receptor (ER)-positive breast cancer patients with metastatic events at both 3 and 5 years expressed lower levels of EI24 compared with their non-metastatic counterparts (Figure 6E, Supplemental Figure 6A). Furthermore, *EI24* gene copy numbers were lower in the group of patients with a metastatic event at 5 years than in patients without a metastatic event (Supplemental Figure 6B). Patients with melanoma who died with metastases at 3 years [35] had lower tumor EI24 expression than patients who were alive at 3 years (Figure 6F). To determine the prognostic importance of EI24 expression, the survival rate of metastatic tumor patients was evaluated using the method described in a previous study [36]. Although there was not a statistically significant correlation between the survival rate of ER-negative breast cancer patients and the expression levels of EI24 and TRAF2 in patients without distant metastasis (Supplemental Figure 6C, 6D), there was a significant correlation between poor survival rate and low EI24 and high TRAF2 expression in ER-positive breast cancer patients (Figure 6G, 6H). Collectively, data from clinical tumor samples reinforce the important role of EI24 in suppressing tumor malignancy.

**Figure 7:**
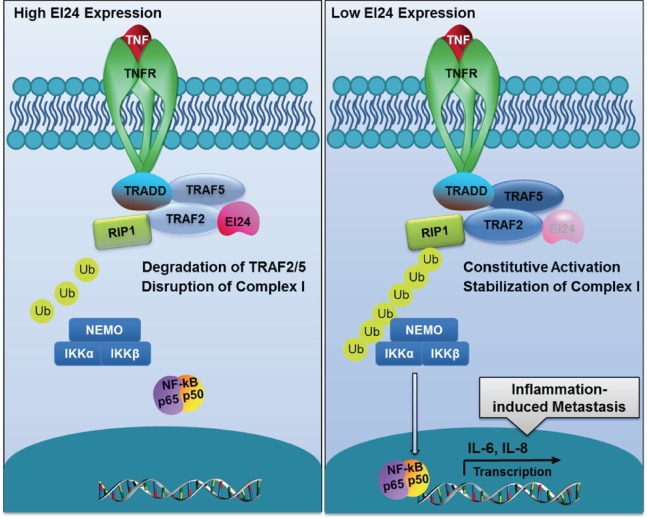
Proposed model of EI24-mediated suppression of tumor progression through the alleviation of NF-κB signaling In the presence of EI24, TRAF2/5 in Complex I are bound with EI24 and subsequently degraded, functioning as a negative regulatory mechanism for the attenuation of downstream NF-κB signaling. When the EI24 level is low, TRAF2 accumulates and promotes Complex I assembly, activation, and the nuclear translocation of NF-κB, resulting in the induction of inflammation-related cytokine expression.

Taken together, these results support a model in which EI24 coordinates EMT and tumor progression through the regulation of TRAF2-mediated NF-κB activity. The pathway identified in this study is illustrated in Figure 7.

## DISCUSSION

In this study, we demonstrated that the reduced expression of a single gene, *EI24,* results in the acquisition of systemic traits that strongly favor tumor progression. Reduction of EI24 expression in tumor cells resulted in the acquisition of various mesenchymal characteristics through the induction of EMT and upregulation of inflammation-related gene expression. We further showed that EI24 promotes tumor progression in a NF-κB-dependent manner by sustaining TNFR1-Complex I activity. In contrast, ectopic expression of EI24 in tumor cells suppressed malignant progression. The multifaceted functions of EI24 in processes required for tumor malignancy make this gene an attractive candidate for therapeutic intervention. Previous studies have shown that the transcriptional activity of NF-κB is required for maintenance of an invasive phenotype in cancers through the upregulation of inflammatory cytokines [37]. Furthermore, NF-κB directly or indirectly regulates mesenchymal markers and EMT-related transcription factors [38]. We found that the induction of EMT upon EI24 ablation was mechanistically similar to the process driven by activation of NF-κB signaling during tumor progression. Based on these observations, we conclude that EI24 inhibits transcriptional activities required for execution of the EMT program in malignant tumor cells.

Since NF-κB is essential for the induction and maintenance of EMT and tumor progression [7], identification of regulators that might be manipulated to decrease NF-κB activity could lead to an ideal therapeutic strategy. Here, we show that the induction of tumor progression by EI24 knockdown involves the activation of NF-κB signaling. A reduction in the level of EI24 induces the expression of the inflammatory cytokines IL-6 and IL-8 that are implicated in tumor malignancy in ZR-75-1 cells, which do not intrinsically express these cytokines. Thus, attenuation of NF-κB signaling by endogenous EI24 is crucial for the suppression of inflammation-induced tumor progression. Since NF-κB is specifically involved in EI24-mediated suppression of EMT without the involvement of other signaling pathways such as PI3K, TGFβ, and p38 MAPK, the therapeutic approach proposed in this study could be a good way to treat tumor progression without severe side effects.

Deubiquitinases such as CYLD or A20 have been proposed as therapeutic targets in inflammatory disorders that involve dysregulation of NF-κB through the regulation of Complex I activity [39]. Moreover, a recent study suggested that degradation of TRAF2 by CHIP results in reduced tumor invasiveness by inactivating NF-κB activity [40]. Here, we demonstrate that lysosome-dependent degradation of the Complex I components TRAF2/5 by EI24 is a novel mechanism for attenuating NF-κB signaling. Thus, as an alternative to CYLD or A20, induction of EI24, which plays a pleiotropic anti-metastatic role, provides a novel molecular target for attenuating NF-κB activation. Consistent with this proposal, a recent study described a therapeutic intervention that targets TRAF protein for the treatment of a cytokine-driven inflammatory disease [41].

Loss of p53 creates an inflammatory microenvironment in an NF-κB-dependent manner to facilitate EMT during tumor progression [42]. Since EI24 contributes to generation of the NF-κB-dependent inflammatory microenvironment and is a transcriptional target of p53, the induction of EMT upon p53 loss may be equivalent to that induced by depletion of EI24 expression. Thus, EI24 may be the effector molecule for p53-mediated suppression of EMT and tumor progression, although further studies are needed to delineate the role of the p53-EI24 axis in these processes.

We used clinical samples from cancer patient-based datasets to investigate the role of EI24 in EMT and tumor progression. Using widely accepted parameters such as changes in mRNA expression and gene copy number variation, our study confirmed that a low level of EI24 correlates with tumor malignancy in humans. Although the current study primarily included melanoma and breast cancers, we also observed decreased EI24 expression in clinical datasets of metastatic ovarian, prostate, and colorectal cancers. Further extensive investigations are needed to elucidate the precise role of EI24 in these cancers.

Collectively, these studies indicate that the expression level of EI24 in malignant tumors could be a useful diagnostic marker. Furthermore, induction of the *EI24* gene may be an effective therapeutic intervention for patients whose tumors carry an intact genomic profile of *EI24*.

## MATERIALS AND METHODS

### Cell culture and generation of stable cell lines

The melanoma B16F1 cells were obtained from the Korean Cell Line Bank (cellbank.snu.ac.kr), and breast cancer NMuMG and MDA-MB-231 cells from the American Type Culture Collection (ATCC; www.atcc.org). B16F10, 4T1 and ZR-75-1 were kindly provided by Drs. Goo Taeg Oh (Ewha University), Sung Hee Baek (Seoul National University), and Jaewhan Song (Yonsei University), respectively. B16F1, B16F10, and MDA-MB-231 cells were cultured in Dulbecco's modified Eagle medium (DMEM, HyClone) supplemented with 10% FBS (HyClone). ZR-75-1 and 4T1 cells were maintained in Roswell Park Memorial Institute Medium (RPMI1640, HyClone) supplemented with 10% FBS. NMuMG cells were grown according to ATCC guidelines.

Human and mouse stable EI24-overexpressing and control (empty vector-harboring) cell lines were generated by transfection with a plasmid containing flag-tagged EI24 or the corresponding empty vector (pcDNA3.1). Clones were selected using G418-containing medium and examined for EI24 expression by immunoblotting. Human and mouse EI24-knockdown stable cell lines were generated as described previously [43].

### Plasmids and reagents

The mouse EI24 constructs were generated as described previously [10]. The human *EI24* ORF was purchased from OriGene and subcloned into the pCS5-3xMyc vector for tagging with myc. TRAF2, TRAF5, RIP1, K63-3x-HA-Ub, and TRADD constructs were generously provided by Dr. Jaewhan Song (Yonsei University). shRNAs against human and mouse EI24 were purchased from Sigma-Aldrich. siRNA sequence against human EI24 is described previously [25]. BAY 11-7082, SB203580, SB435142, and Wortmannin were purchased from Tocris.

### Immunoblotting, immunoprecipitation, and antibodies

Protein extraction and immunoprecipitation were performed as described previously [44]. Exposures were acquired using a LAS-3000 Imager (Fujifilm). The following antibodies were used: anti-EI24 [44], anti-E-cadherin (Cell Signaling Technology, #4065), anti-β-catenin (BD Transduction, #610153), anti-γ-catenin (Cell Signaling Technology, #2309), anti-vimentin (Abcam, #ab8978), anti-TRAF2 (Cell Signaling Technology, #4724), anti-RIP1 (BD Transduction, #610459), anti-TRADD (Santa Cruz Biotechnology, #sc-7868), anti-TNFR1 (R&D systems #AF225 for IP; Santa Cruz Biotechnology #sc-8436 for IB), anti-poly (ADP-ribose) polymerase (PARP; Cell Signaling Technology, #9542), anti-p65 (Cell Signaling Technology, #8242), anti-phospho (p)-p65 (Cell signaling Technology #3033), anti-IKKβ (Cell Signaling Technology, #2370), anti-p-IKKα/β (Cell Signaling Technology, #2697), anti-IκBα (Cell Signaling Technology, #4814), anti-p-IκBα (Cell Signaling Technology, #2859), anti-MYC (Cell Signaling Technology, #2276), anti-HA (Santa Cruz Biotechnology, #sc-7392), and anti-FLAG (Sigma, #F3165).

### Ubiquitination assay

Cells were harvested in PBS containing 2 mM N-ethylmaleimide (NEM) and lysed in Tris-buffered saline (TBS) containing 1% SDS and 20 mM NEM. The lysate was boiled, sonicated, and centrifuged at 14,000 g for 15 min. The supernatant was diluted in NP-40 buffer containing 2 mM NEM, and immunoprecipitation was carried out using standard methods.

### RNA isolation and real-time qPCR

Total RNA was prepared with Trizol reagent (Invitrogen). One microgram of total RNA was reverse-transcribed to cDNA using the Superscript III First-Strand Synthesis System with oligo-dT primers (Invitrogen). Real-time qPCR was performed using IQ SYBR Green SuperMix on an iQ5 Real-Time PCR System (Bio-Rad), with each sample measured in triplicate. Relative gene expression values were calculated using the iQ5 optical system software (Bio-Rad) after normalization to the expression levels of *GAPDH*. The sequences of primers were designed using IDT SciTools (www.idtdna.com) and are as follows: human *EI24* forward, 5ʹ-AATGCACCAGCGGTTGTCTAA-3ʹ; human *EI24* reverse, 5ʹ-GATAGAGAAAAGGCAGCCACTGA-3ʹ, human *CDH1* forward, 5ʹ-CTACGGAGGAGAACGGTGGT-3ʹ; human *CDH1* reverse 5ʹ-GGCTCAAATCAAAGTCCTGGT-3ʹ. Primer sequences for assessment of human *IL6* and *IL8* [45] and other EMT-related genes [46] were described previously.

### Migration and invasion assay

Migration assays were performed using the Transwell Permeable Support System (Corning Costar) with a fibronectin pre-coated polycarbonate membrane (8 μm pore size). The invasion assay was performed with the BD BioCoat Matrigel Invasion Chamber (BD Bioscience). For both assays, cancer cell lines cultured in serum-free medium were seeded on the top of the upper chamber, while the bottom chambers were filled with regular culture media. Cells that had moved and were attached to the lower side of the membrane were fixed and cells remaining in the upper chamber were removed using a cotton swab. Cells that had migrated to the lower side of the membrane were stained with crystal violet and assessed the migration and invasion abilities of the cells.

### Hanging drop assays

Approximately 25,000 single cells were dropped onto the lid of a 60-mm culture dish (Nunc). The corresponding bottom surfaces of each dish contained the appropriate culture media to maintain humidity. The images of drops were captured with an inverted microscope (×4 magnification; Nikon). At least five independent drops were analyzed for each experiment. The area of cell clusters was determined using the NIS-Element Analysis Tool for the Nikon microscope (Nikon).

### Anoikis assay

Equal volumes and numbers of cells were maintained in ultra-low-attachment plates (Corning Costar) to induce anoikis. Live/dead cells were scored by manual counting following Trypan Blue staining (Sigma-Aldrich) at the indicated time points according to the manufacturer's instructions.

### 3D Matrigel culture

ZR-Ctrl and ZR-shEI24 cells in growth medium containing 2% Matrigel were seeded on 8-well chamber slides (BD Biosciences) pre-coated with Matrigel. Cell morphology was assessed and the cells were photographed under a microscope (Nikon) over time until invasive acini appeared.

### Immunocytochemistry

Cells were seeded on 4-or 8-well chamber slides (BD Bioscience) at a density of 3 × 10^4^ cells per slide. The cells were fixed with 4% paraformaldehyde, permeabilized with 0.1% Triton X-100 (Amersham), and then blocked with 2.5% bovine serum albumin (Sigma-Aldrich) for 30 min and incubated with primary antibodies overnight. After staining with Alexa Flour 488-or Alexa Flour 568-conjugated secondary antibodies (Invitrogen) and extensive washing, the slides were mounted with ProLong Gold antifade reagent in the presence of DAPI for visualization of the nuclei (Invitrogen). Images were captured using a fluorescence microscope (Nikon) or a confocal microscope (Zeiss). The following antibodies were used for immunocytochemistry: anti-Paxillin (BD Transduction, #610569), anti-E-cadherin (Cell Signaling Technology, #4065), anti-Vimentin (Abcam, #ab8978), anti-Rhodamine phalloidin (Invitrogen, R415), anti-MYC (Cell Signaling Technology, #2276), and anti-FLAG (Sigma, #F3165).

### Luciferase reporter assay

Luciferase reporter assays were performed with the Dual-Luciferase Reporter Assay System (Promega) according to the manufacturer's instructions. Cells were transfected with 4x-κB-luc and pRL-tk, the internal control encoding Renilla luciferase, using lipofectamine 2000 (Invitrogen). After 24 hours, the luciferase activity was measured using a luminometer (Turner Biosystems, Promega) and the relative luciferase reporter activity was calculated after normalization of the firefly luciferase reporter activity to the Renilla luciferase activity.

### Animals and in vivo metastasis assay

Wild-type C57BL/6NTac mice were purchased from Taconic (USA). Mice were housed in a specific pathogen-free facility at Yonsei University Laboratory Animal Research Center with a 12-hour dark-light cycle and handled according to the Institutional Animal Care and Use Committee standards. For analysis of in vivo metastasis of melanoma cells, wild-type C57BL/6NTac mice (6-8 weeks old) were injected with 3 × 10^4^ cells resuspended in PBS via the tail veins and sacrificed after 2-3 weeks. Metastatic nodules in the lung and liver were counted under a microscope (Nikon).

### Microarray and Oncomine analyses

Gene expression profiling studies of ZR-shEI24 and control cells were performed using the GeneChip human exon 1.0 st array (Affymetrix). RNA samples were prepared using the RNeasy Mini Kit (QIAGEN), and microarray studies were carried out following standard Affymetrix protocols of DNALink, Inc. (http://www.dnalink.com). The expression microarray data are deposited in the GEO database with the accession number GSE52508.

To examine *EI24* gene expression and copy number variation in human patients, we used several datasets from the Oncomine Premium Edition Database (Compendia Biosciences, USA; www.oncomine.org). Statistical analysis of differences in expression was performed using ONCOMINE algorithms as described previously [29].

### Gene Set Enrichment Analysis (GSEA)

Version 2.0.10 of the GSEA desktop application was provided by the Broad Institute of MIT and Harvard University (www.broad.mit.edu/gsea) and used as described previously [47]. Gene signatures were obtained from MSigDB v3.1 or from published gene lists as described in the main text. The parameters and presentations of GSEA were processed as described previously [42].

### Statistical analysis

Data are presented as mean ± S.D. For statistical analysis we used the GraphPad Prism software. The statistical analyses were conducted using the unpaired t-test unless otherwise stated. Values of p <0.05 were considered statistically significant.

## Supplementary Figures and Tables




