# Supplementary material for: EI24 regulates epithelial-to-mesenchymal transition and tumor progression by suppressing TRAF2-mediated NF-κB activity
Source: Oncotarget. 2013 Nov 17;4(12):2383–96. doi: 10.18632/oncotarget.1434 (PMC3926834; doi:10.18632/oncotarget.1434)
Supplement: Supplementary file 1 [file oncotarget-04-2383-s001.pdf]

EI24 regulates epithelial-to-mesenchymal transition and tumor progression by suppressing TRAF2-mediated NF-κB activity - Choi et al

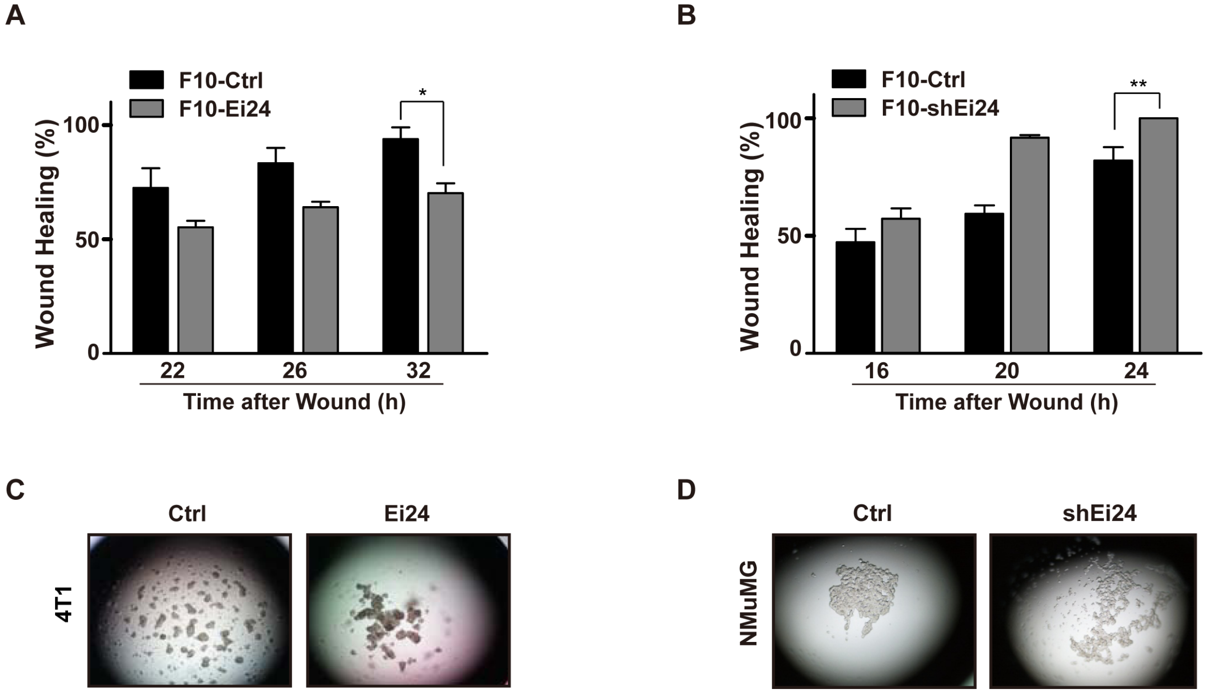

Supplemental Figure 1: Regulation of EMT-prerequisite functions by EI24. (A) and (B) Wound-healing in cultured F10-Ctrl and F10-Ei24 cells at 22-32 h (*p <0.0001, a) and 16-24 h (**p <0.005, b). Data shown are the mean ± S.D. from seven fields. (C) and (D) Hanging drop assay with 4T1-Ctrl and 4T1-Ei24 (C) and NMu-Ctrl and NMu-shEi24 (D) cells.

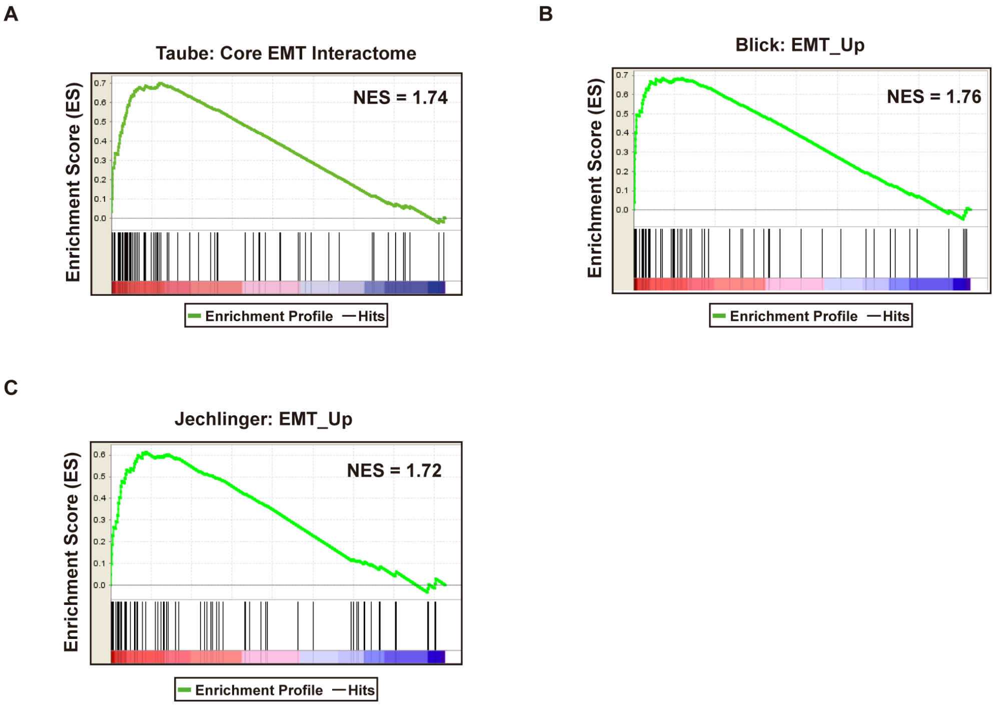

Supplemental Figure 2: Gene expression profiles upon EI24 knockdown are characteristic of EMT. (A) GSEA showing enrichment of a mesenchymal gene signature in ZR-shEI24 cells. NES, normalized enrichment score; p <0.001. (B) and (C) GSEA showing enrichment of gene signatures that are upregulated during the EMT process from two independent studies of the Blick dataset (B) and Jechlinger dataset (C). Both plots were obtained by comparing genes expressed in ZR-75-shEI24 with those expressed in control cells. NES, normalized enrichment score; p <0.001.

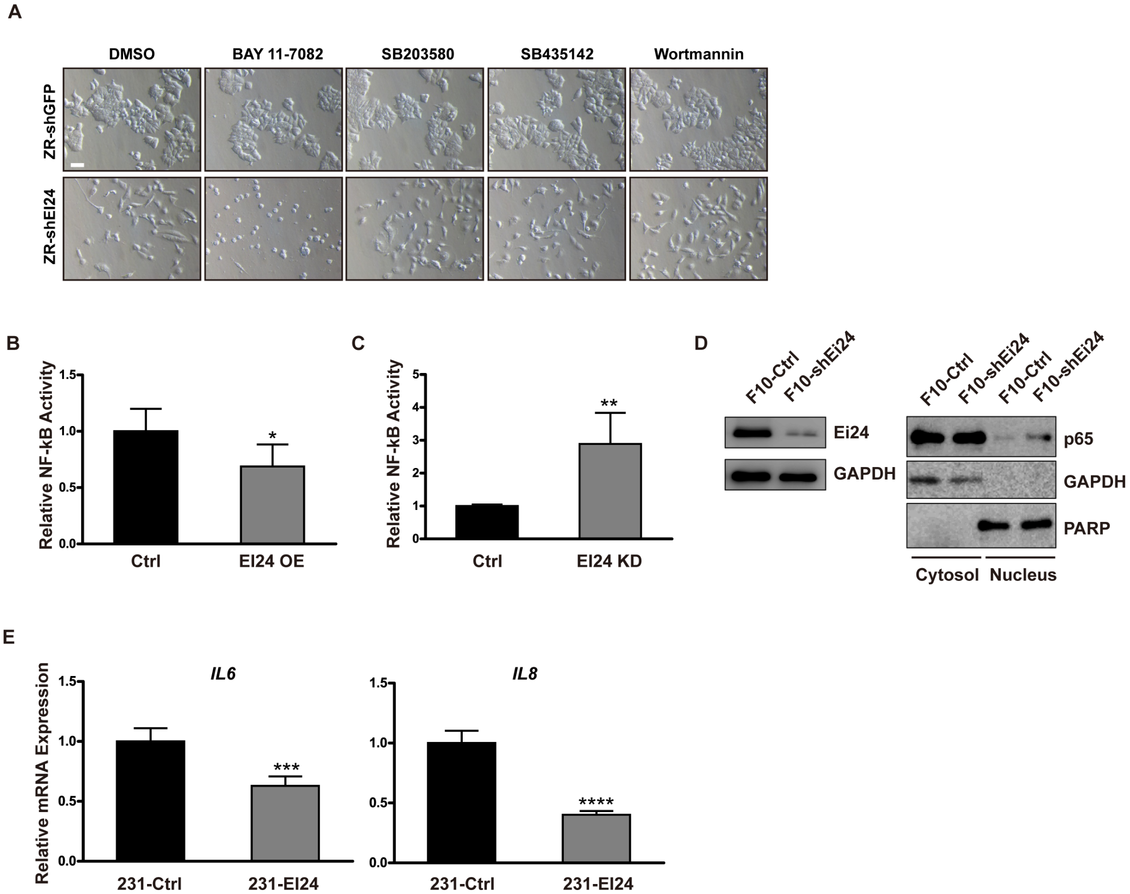

Supplemental Figure 3: EI24-mediated regulation of NF-κB induces morphological changes and expression of downstream transcriptional targets. (A) Morphology of cells after treatment with vehicle (DMSO) or inhibitors of NF-κB (BAY 11-7082), p38 MAPK (SB203580), TGFβ (SB435142), or PI3K (Wortmannin) signaling pathways. Scale bar, 100 µm. (B) and (C) NF-κB reporter activity in control, EI24-overexpressing (n = 12; B), and EI24-knockdown (n = 3; C) HeLa cells. *p <0.001, **p <0.05. (D) Immunoblot analysis of p65 expression in nuclear and cytosolic fractions from control and F1-shEi24 cells. (E) Real-time qPCR analysis of mRNA expression levels of the indicated genes in control and 231-EI24 cells. All data shown are mean ± S.D. for experiments performed at least twice, with each sample assayed in triplicate. P values were calculated by unpaired t-test using GraphPad Prism software. *p <0.001, ***p <0.0001, ****p <0.0005.

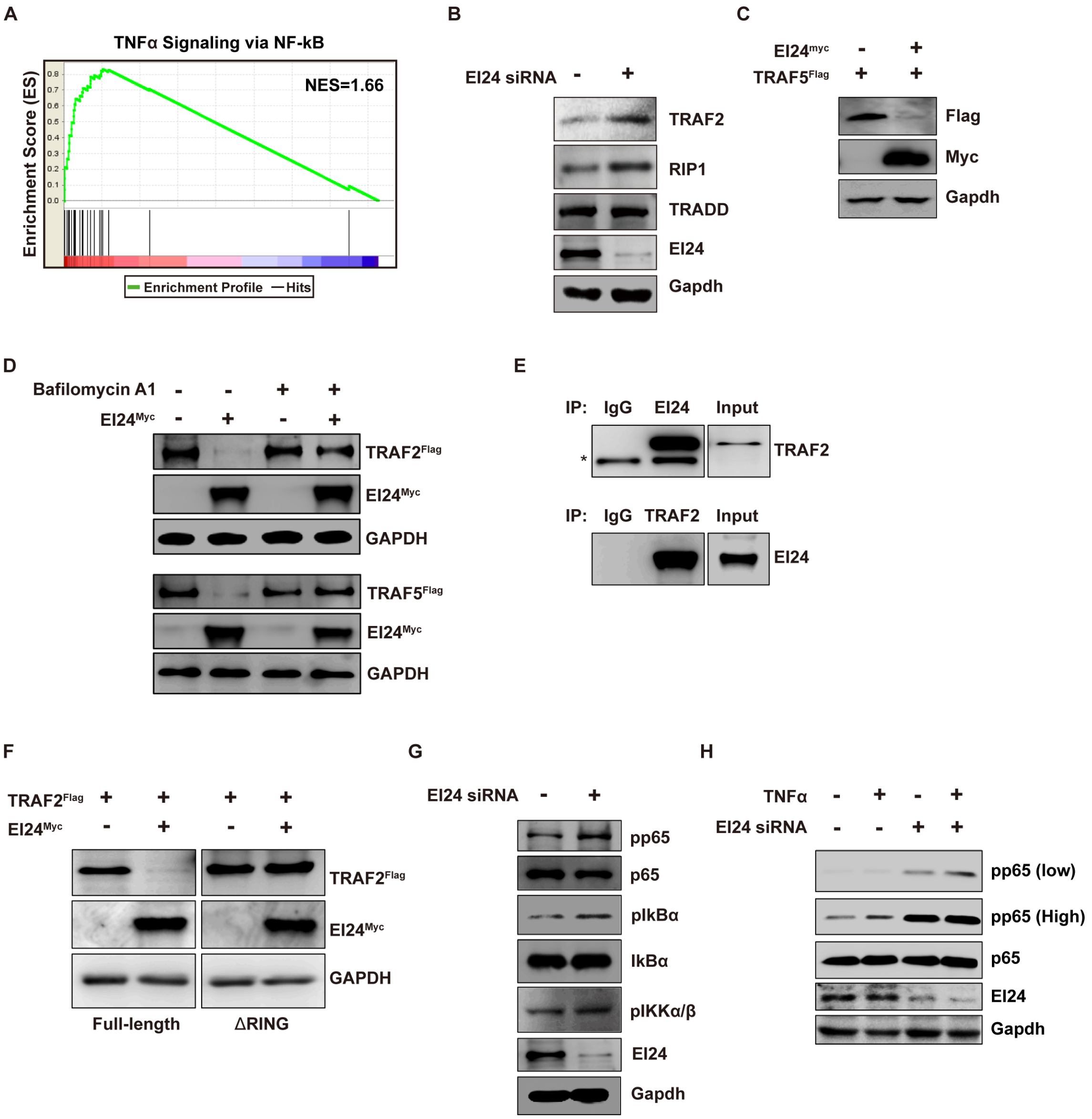

Supplemental Figure 4: EI24 regulates Complex I signaling. (A) GSEA showing gene signatures of NF-κB-mediated TNFα signaling (p <0.001). (B) Immunoblot analysis of endogenous protein levels of Complex I components in HeLa cells with and without knockdown of EI24. (C) Immunoblot analysis of EI24-mediated TRAF5 degradation. (D) EI24-mediated degradation of TRAF2 and TRAF5 was rescued by treatment with the lysosome inhibitor bafilomycin A1 (10 μg/ml, 12 hours). (E) Binding between endogenous EI24 and TRAF2. Extracts were immunoprecipitated as indicated above the panels and immunoblotted with antibodies against TRAF2 (upper panel) or EI24 (lower panel). *IgG heavy chain. WCL, whole cell lysate. (F) Immunoblot analysis showing that the RING domain of TRAF2 is required for its degradation in 293T cells overexpressing EI24. (G) Immunoblot analysis showing activation of downstream signaling molecules of Complex I upon EI24 knockdown. (H) p65 activation in HeLa cells upon knockdown of EI24 with or without TNFα (10 μg/ml).

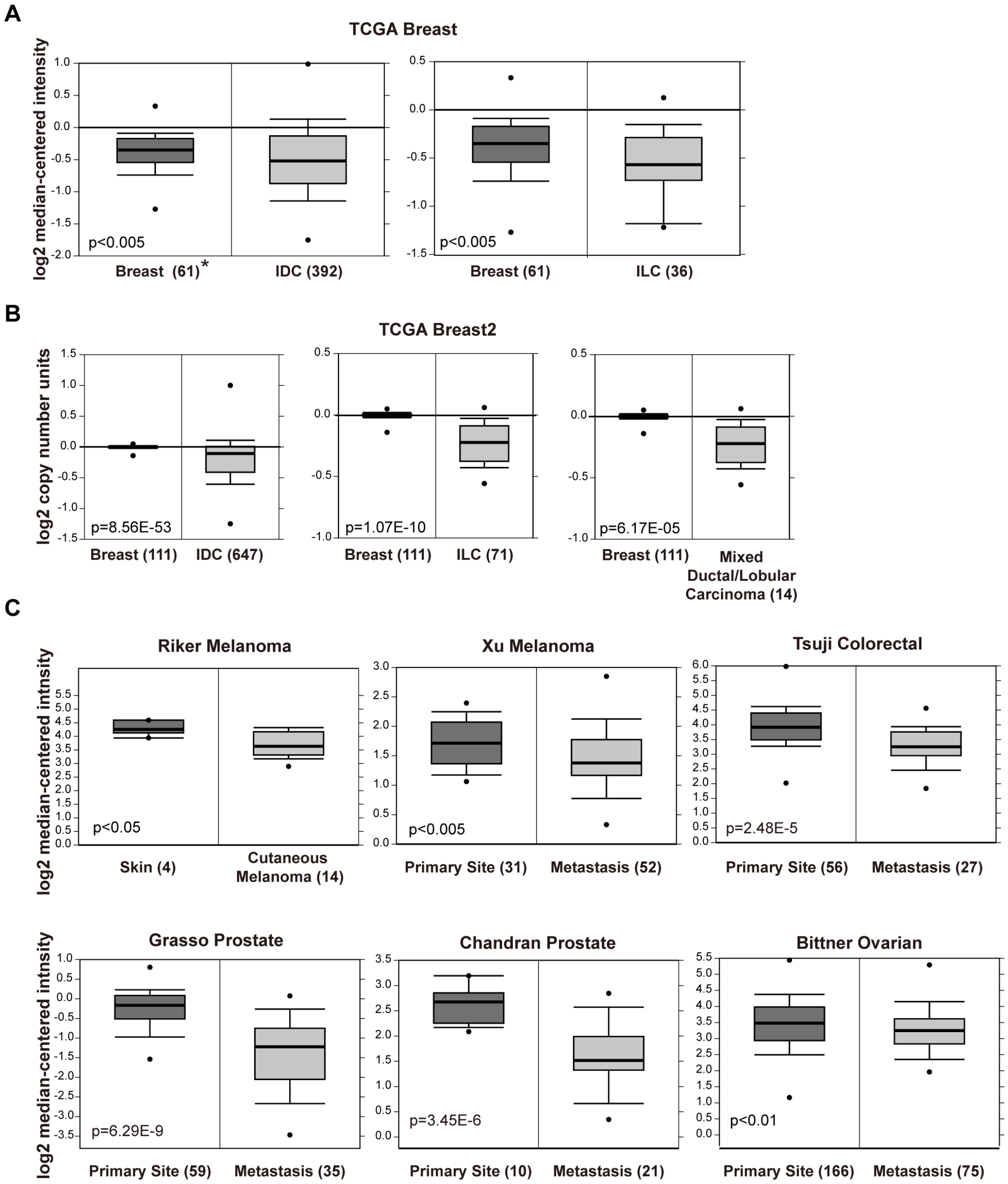

Supplemental Figure 5: Loss of EI24 results in metastatic progression in human patients. (A) Box plots showing EI24 gene expression in normal breast tissue and in IDC and ILC patients within the TCGA dataset. (B) Box plots showing EI24 gene copy numbers in normal breast tissue and in IDC and ILC patients within the TCGA2 dataset. (C) Box plots showing EI24 gene expression in various metastatic tumors. *Numbers in parentheses indicate number of samples included in the study.

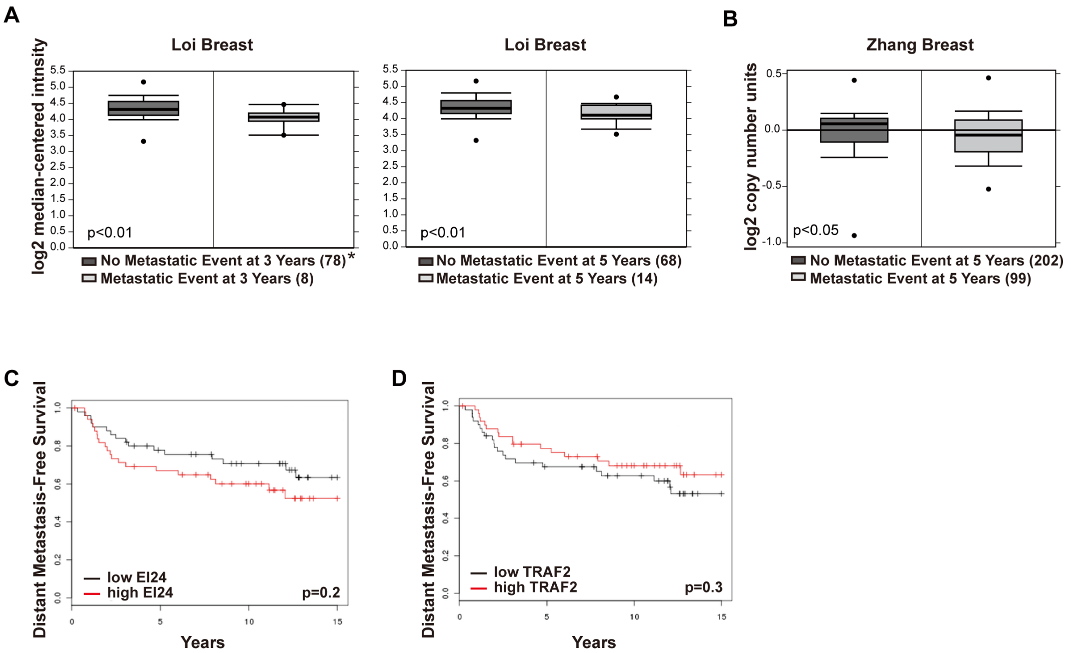

Supplemental Figure 6: Correlation between EI24 expression level in clinical samples and clinical outcomes. (A) EI24 gene expression in breast cancer patients with clinical outcomes of one or more metastatic events at 3 and 5 years. (B) EI24 copy numbers in breast cancer patients with one or more metastatic events at 5 years. (C) and (D) Kaplan-Meier plots of distant metastasis-free survival of breast cancer patients. ER-negative patients (n = 87) were evaluated from 2011 version of database and categorized by median expression of EI24 (208289_s_t, C) or TRAF2 (204413_at, D). The p-values were calculated using the log rank test. *Numbers in parentheses indicate number of samples included in the study.
